# Between Healthcare Practitioners and Clergy: Evangelicals and COVID-19 Vaccine Hesitancy

**DOI:** 10.3390/ijerph191711120

**Published:** 2022-09-05

**Authors:** Jeanine P. D. Guidry, Carrie A. Miller, Paul B. Perrin, Linnea I. Laestadius, Gina Zurlo, Matthew W. Savage, Michael Stevens, Bernard F. Fuemmeler, Candace W. Burton, Thomas Gültzow, Kellie E. Carlyle

**Affiliations:** 1 Media + Health Lab, Robertson School of Media and Culture, Virginia Commonwealth University, 901 W. Main Street, Suite 2216, Richmond, VA 23284, USA; 2 Department of Public Relations, STEM Translational Communication Center, College of Journalism and Communications, UF Health Cancer Center, University of Florida, Gainesville, FL 32611, USA; 3 School of Data Science, Department of Psychology, University of Virginia, Charlottesville, VA 22904, USA; 4 Joseph J. Zilber School of Public Health, University of Wisconsin-Milwaukee, Milwaukee, WI 53205, USA; 5 Gordon-Conwell Theological Seminary, Hamilton, MA 01982, USA; 6 School of Communication, San Diego State University, San Diego, CA 92182, USA; 7 Section of Infectious Diseases, Department of Medicine, West Virginia University School of Medicine, Morgantown, WV 26506, USA; 8 Massey Cancer Center, Department of Health Behavior and Policy, School of Medicine, Virginia Commonwealth University, Richmond, VA 23219, USA; 9 Sue & Bill Gross School of Nursing, University of California at Irvine, Irvine, CA 92697, USA; 10 Department of Work and Social Psychology, Maastricht University, 6200 MD Maastricht, The Netherlands; 11 Department of Health Behavior and Policy, School of Medicine, Virginia Commonwealth University, Richmond, VA 23219, USA

**Keywords:** COVID-19 vaccine, evangelicals, health belief model, healthcare providers, vaccine hesitancy

## Abstract

Evangelical Christians are among the most hesitant to get the COVID-19 vaccine. This study examined the extent to which COVID-19 vaccination uptake among Evangelicals is explained by demographic characteristics, Health Belief Model constructs, and faith-based support factors. Survey research firm Qualtrics recruited 531 U.S. adults and conducted a survey to explore predictors of COVID-19 vaccine uptake among people who self-identified as Evangelicals in September 2021. A logistic regression showed that those reporting high perceived benefits of the COVID-19 vaccine were more likely to be vaccinated, while those reporting high perceived barriers were less likely to be vaccinated. Those whose healthcare provider asked them about the vaccine were more likely to be vaccinated than those whose healthcare provider did not ask. Finally, while those who reported information seeking from religious leaders were less likely to be vaccinated, those who reported more faith-based support for vaccination were more likely to be vaccinated. In addition to beliefs about benefits and barriers to vaccination, the role of healthcare providers and clergy were important factors influencing vaccination status. Intervention efforts that capitalize on partnerships between health providers and clergy in supportive congregations may be able to reach undecided Evangelicals.

## 1. Introduction

Successful development and deployment of COVID-19 vaccines is key to controlling the COVID-19 pandemic. In spite of COVID-19 vaccine availability since late 2020, vaccine hesitancy is a significant obstacle to achieving widespread vaccination [1]. As of April 2022, 17% of U.S. Americans still indicated that they definitely did not plan to get vaccinated [2,3]. Vaccine hesitancy has risen so substantially that the World Health Organization (WHO) now considers it a major threat to global health [4].

Those identifying as Evangelical Christians are among the groups that, so far, have been more hesitant to get the COVID-19 vaccine in the U.S. [5,6]. As of May 2022, White Evangelical Christians represented almost a third of unvaccinated adults in the U.S. [3]. Frequently mentioned reasons for COVID-19 vaccine hesitancy in general are that these are not safe and that the COVID-19 vaccines were developed too quickly, as well as needle phobia [7,8,9]. Common reasons for vaccine hesitancy specifically relevant among Evangelical Christians are that vaccines interfere with divine providence, and that vaccine development and manufacturing involves aborted stem cells [10,11]. Evangelicals are also somewhat more likely than those of other religious affiliations to indicate that science sometimes conflicts with their own religious beliefs. Importantly, studies demonstrate that religious leaders and shared religious identities can have a significant effect on vaccination decisions, both to vaccinate [12,13], as well as to discourage vaccination [14]. With Pew Research Center reporting 29% of Americans self-identify as Evangelicals [15], better understanding COVID-19 vaccine hesitancy in this segment of the population is of great relevance to ending the pandemic.

The Health Belief Model (HBM), long used for understanding vaccination behaviors [16], is useful for understanding COVID-19 vaccine uptake behavior. Its constructs, as applied to COVID-19 and COVID-19 vaccines, are perceived severity of and perceived susceptibility to COVID-19, perceived benefits of and barriers to obtaining a COVID-19 vaccine, self-efficacy to overcome vaccination barriers, and cues to action to get a COVID-19 vaccine. The research questions for this study are, therefore:

RQ1: What demographics, Health Belief Model constructs, and faith-based factors among self-identified Evangelicals predict those who have received the COVID-19 vaccine vs. and those who have not?

RQ2: What demographics, Health Belief Model constructs, and faith-based factors among self-identified Evangelicals predict differing levels of vaccine hesitancy among those who have not yet received the COVID-19 vaccine?

## 2. Materials and Methods

Using panel research firm Qualtrics, a survey of 531 English-speaking U.S. adults between the ages of 18 and 65 who self-identified as Evangelicals was conducted to explore the relationships between demographics and psychosocial predictors derived from the HBM of COVID-19 vaccine uptake among Evangelicals. Participants completed the survey in September 2021. To meet inclusion criteria, once participants identified as Christian in the screening question section of the survey, they answered the question: “If you identify as Christian, which of the following categories applies to you?”. Answer options were “Mainline Protestant (such as Presbyterian, Methodist, Lutheran, Episcopalian),” “Evangelical Protestant (such as Assemblies of God, Pentecostal, Southern Baptist, Nondenominational charismatic),” “Historically Black Protestant,” “Catholic,” or “other”. Only the respondents who self-identified as “Evangelical Protestant” were then part of the actual study sample (those who answered all study questions). The study was approved by the Institutional Review Board at a large public research university in the Mid-Atlantic U.S. (ID number: HM20025520).

### 2.1. Measures

**Demographics.** Variables included age, gender, race/ethnicity, education, health insurance status, and rurality.

**Faith-Based Variables.***Religious leader information seeking* for COVID-19 vaccine uptake advice was measured using one item: “When deciding whether to get a COVID-19 vaccine, how much did you turn to religious leaders for information,” with answer options “Not at all,” “A little,” “Some,” and “A lot.” *Faith-based support for vaccination* was measured with four questions, all with the response options of “Much less likely,” “Somewhat less likely,” “Would make no difference,” “Somewhat more likely,” and “Much more likely.” The stem of these questions was “Would each of the following make you more likely to get vaccinated for COVID-19, less likely to get vaccinated for COVID-19, or would it make no difference,” with questions: “A religious leader you trust got the vaccine,” “You could get the vaccine at a nearby religious congregation,” “A religious leader you trust encouraged you to get the vaccine,” and “Your religious community provided assistance in getting an appointment to get the vaccine.” Cronbach’s alpha for these four variables was 0.84; therefore, the mean was used in the analyses (see Table 1).

**Health Belief Variables.** Participants responded to each of the items described below using a seven-point Likert scale that ranged from “strongly disagree, 1” to “strongly agree, 7” except for the question about the certainty of access to the vaccine in the self-efficacy domain, which used a seven-point Likert-type scale ranging from “very uncertain, 1” to “very certain, 7.” These variables were based on pandemic vaccine scales developed by Myers and Goodwin [17] (see Table 1).

*Perceived severity* of COVID-19 was measured using three items (e.g., “I will be very sick if I get COVID-19”). Cronbach’s alpha for items on the scale was 0.76. The mean of the scale items was used in the analyses.

*Perceived susceptibility* to COVID-19 was measured using three items (e.g., “My chance of getting COVID-19 in the next few months is high”). Cronbach’s alpha for items on the scale was 0.81. The mean of the scale items was used in the analyses.

*Perceived benefits to vaccination* were measured using four items focused on the benefits of a COVID-19 vaccine (e.g., “Getting a COVID-19 vaccination helped me feel less worried about getting COVID-19”). Cronbach’s alpha for these items was 0.82. The mean of the scale items was used in the analyses.

*Perceived barriers to vaccination* were measured using 10 items (e.g., “Scientists did not have enough time to assess the possible risks of a COVID-19 vaccine, “I am concerned about the side effects of a COVID-19 vaccination,” and “I am scared of needles”). Cronbach’s alpha for these items was 0.80. The mean of the scale items was used in the analyses.

*Self-efficacy* was measured with three items (e.g., “How certain are you that you could get a COVID-19 vaccination?” with responses ranging from “very uncertain” to “very certain”). Cronbach’s alpha for items on the scale was 0.77. The mean of the scale items was used in the analyses.

*Cues to action* was measured using one item, “Has your healthcare provider talked to you about getting the COVID-19 vaccine?”, with “yes” and “no” as response options.

**Vaccination Uptake.***COVID-19 vaccine uptake* was measured using one item with the following four options: “I already received one or more doses of the COVID-19 vaccine,” “I am planning to get the COVID-19 vaccine as soon as possible,” “I am undecided about whether I will get the COVID-19 vaccine,” and “I will **not** get the COVID-19 vaccine.” This measure was collapsed into a discrete “vaccinated/not vaccinated” dependent variable for use in a logistic regression and used in its original form for the multinomial regression. The dichotomous vaccination status variable was collapsed as follows: “vaccinated” meant the respondent at least received one dose of a COVID-19 vaccine. The other three question options, “I am planning to get vaccinated,” “I am undecided,” and “I will not get the COVID-19 vaccine” were all collapsed into “not vaccinated.”

### 2.2. Statistical Approach

Given that we were interested both in predictors of vaccination status (e.g., vaccinated/not vaccinated) and in predictors differentiating levels of vaccine hesitancy (e.g., planning vs. unwilling and undecided vs. unwilling), we ran three binary logistic regressions. Following descriptive analyses performed using SPSS 28.0, a binary logistic regression analysis was used to explore how demographic characteristics, health belief variables related to the COVID-19 vaccine, and faith-based vaccination support variables predicted COVID-19 vaccine uptake status as a binary dependent variable. Then, two binary logistic regressions were used to explore differences between the unwilling group and the other two (planning and undecided) types of non-vaccinated respondents. These three logistic regressions were more parsimonious than one overall multinomial logistic regression (with numerous follow-up binary logistic regressions) examining predictors of all vaccination types and also had the added benefit of examining predictors of participants who had vs. had not been vaccinated. In order to control for all demographics and theoretically relevant variables, no approach entailing backward elimination of predictors was employed, and all predictor variables—no matter their statistical significance level—were retained in the regression models. While in many cases, predictor variables are added to a model based on their statistical significance and theoretical plausibility, we used the HBM as a framework; therefore, we had a specific theory about the respective predictors included. Given that COVID-19 vaccine hesitancy in Evangelicals has not yet been examined in the literature, we did not have a compelling pre-existing theory, by contrast, about which demographic predictors in this specific sample might play an important role. As a result, all variables, even when ultimately non-significant, were included in the model; the initial models, therefore, were also the final retained models.

## 3. Results

Of the 531 survey participants, 64.8% identified as women and 35.2% as men, and 82.1% reported being White, 11.5% Black, 4.0% Hispanic or Latinx, and 2.4% other. Considering these ratios, we collapsed the race variable into White (82.1%) and non-White (17.9%), given that this breakdown is consistent with the national trends in the U.S. where the majority of Evangelical Christians are White (76%) [18]. The mean age of participants was 51.2 (*SD* = 16.52). Of the total sample, 90.4% reported having health insurance. Finally, 25.2% reported having completed some high school, 39.2% some college, and 35.6% either a bachelors or graduate degree; moreover, 32.4% reported living in a rural area, 31.1% in an urban area, and 36.5% in a suburban area (Table 2).

### 3.1. Vaccination Uptake

Of the total sample, 40.5% of respondents reported they had already gotten at least one COVID-19 vaccine dose (designated as “Vaccinated”); 19.6% said they were planning to get the vaccine (designated as “Planning”); 20.3% reported to be undecided (designated as “Undecided”); and 19.6% reported they would definitely not get the COVID-19 vaccine (designated as “Definitely will not get vaccinated”).

### 3.2. Predictors of COVID-19 Vaccine Uptake

A binary logistic regression was performed to ascertain the effects of age, gender, health insurance, parent status, education, race/ethnicity, rurality, HBM constructs, faith-based support for COVID-19 vaccination, and religious leader information seeking on the likelihood that participants would have received the COVID-19 vaccine (vaccinated vs. not vaccinated). The assumption of linearity was assessed and met via the inspection of scatter plots among predictors, generally with oval or elliptical shapes. Multicollinearity was assessed using Variance Inflation Factor (VIF) values and was found not to be violated, with all VIF values being under 2. Finally, there were 11 (less than 2% of the sample) multivariate outliers identified with the calculation of the Mahalanobis distance, which were retained in the analysis considering their likely small influence on the model given their scarcity. The logistic regression model was statistically significant, χ^2^(17) = 329.520, *p* < 0.001. The model correctly classified 84.9% of cases (Nagelkerke pseudo R^2^ = 0.712). Of the five predictor variables, eight were statistically significant.

For participant demographics, the results indicated (Table 3) that older respondents were more likely to be vaccinated than younger respondents (specifically, per additional year of life, the chances of being vaccinated increased by 4%) (*p* < 0.001). In addition, compared with respondents without children under 18 living at home, parents of children under 18 were almost three times less likely to be vaccinated (*p* = 0.009). In addition, compared with those living in rural areas, respondents living in suburban areas were over twice as likely to have received the COVID-19 vaccine (*p* = 0.019). Regarding the health belief variables, those who reported high perceived benefits of the COVID-19 vaccine were more likely to be vaccinated, while those who reported high perceived barriers to the COVID-19 vaccine were less likely to be vaccinated (*p*’s < 0.001). In addition, those whose healthcare provider asked them about the vaccine were twice as likely to be vaccinated than those whose healthcare provider did not ask (*p* = 0.027). Finally, for faith-based variables, while those who reported information seeking from religious leaders (*p* < 0.001) were less than half as likely to be vaccinated, those who reported more faith-based support for vaccination (*p* = 0.007) were more likely to be vaccinated.

### 3.3. Differences among the Non-Vaccinated Groups

Two additional binary logistic regression were used to examine the effects of demographics, health beliefs, and faith-based variables among the three non-vaccinated groups (excluding those who were already vaccinated) and comparing those who were unwilling to get vaccinated to the other two groups (undecided and planning). The predictors’ odds ratios and 95% CIs for the “undecided” vs. “will not get vaccinated” and “planning” vs. “will not get vaccinated” logistic regressions are presented in Table 4.

The first binary logistic regression compared those who were undecided about getting the vaccine compared to those who did not intend to get the vaccine. Compared with those who had a bachelor’s degree or higher, those with some high school or a high school diploma were almost three times less likely to be undecided about getting the vaccine than to be unwilling to get the vaccine (*p* = 0.046). In other words, those who were unvaccinated and who had higher levels of education were more likely to be unwilling to get the COVID-19 vaccine vs. undecided. Regarding the health belief variables, participants who reported higher perceived benefits of the COVID-19 vaccines (*p <* 0.001), as well as lower perceived barriers to the vaccine (*p* < 0.001), were more likely to report being undecided about getting the vaccine than being unwilling. No faith-based variables emerged as significant when comparing those who were undecided about getting the vaccine and those who did not intend to get the vaccine.

The next binary logistic regression compared those who were planning to get the vaccine and those who were unwilling. Compared with those who had a bachelor’s degree or higher, those with some high school or a high school diploma were again more likely to be planning to get the vaccine than to be unwilling (*p* = 0.002). Compared with those living in urban areas, those who lived in suburban areas were less likely to be planning to get the vaccine than to be unwilling (*p* = 0.029).

Regarding the health belief variables, participants who reported higher perceived benefits of the COVID-19 vaccines (*p* < 0.001), lower perceived barriers to getting the vaccine (*p* < 0.001), and higher perceived susceptibility to COVID-19 (*p* = 0.002) were more likely to report planning to get the vaccine than being unwilling. Regarding cues to action, compared with those whose healthcare provider spoke to them about getting the COVID-19 vaccine, those whose healthcare provider did not speak to them about getting the vaccine were less likely to report planning to get the vaccine than to be unwilling (*p =* 0.023). In other words, if a healthcare provider had spoken to the respondent about the COVID-19 vaccine, they were more likely to be in the unwilling group. When considering faith-based variables, participants who reported placing a higher value on faith-based support for vaccination were more likely to report planning to get the vaccine than to be unwilling (*p* = 0.035). Advice from religious leaders did not emerge as significant when comparing those who reported planning to get the vaccine versus those having decided not to get the vaccine.

## 4. Discussion

Although COVID-19 vaccination uptake has been widely previously studied, there are still large gaps in the literature about factors that could potentially assist public health officials in reaching and communicating with hesitant groups, such as Evangelical Christians, to consider vaccinations. To that end, this study contributes by revealing demographic characteristics, health beliefs, and faith-based predictors of COVID-19 vaccination uptake among Evangelical Christians in the United States. The results here obtained suggest that we must work to reach Evangelicals who reside in rural areas, are younger, and/or who have children. Communication with them should focus on health beliefs that bolster the perceived benefits of the vaccine, while simultaneously adequately addressing perceived barriers to vaccination. Importantly, the pattern of findings in the present study suggests that addressing these health beliefs might be best accomplished through a careful collaboration between public health officials, healthcare providers, and religious leaders.

Demographically, older Evangelical Christians, as well as those living in suburban areas, were more likely to have gotten the COVID-19 vaccine than their rural counterparts. This is consistent with flu vaccine studies, where older participants were more likely to get the flu vaccine and those living in rural areas were less likely to get the flu vaccine [19,20]. The results also demonstrated that the parents of children under 18 were less likely to be vaccinated than respondents who either had no children or no children under 18 still living at their home. A recent Kaiser Family Foundation poll [21] found that unvaccinated parents were also strongly opposed to their child getting the COVID-19 vaccine. This finding, then, is concerning, given the important need to reach children in the U.S. vaccination effort, since 22% (74 million) of the U.S. population is under 18 years of age, and evidence has emerged indicating that children and adolescents play a role in spreading COVID-19. For example, high school outbreaks have been reported worldwide [22] and small social gatherings, especially childhood birthdays, have been associated with increased COVID-19 infections within households [23]. When it came to comparing unvaccinated groups, those with higher levels of formal education were more likely to be unwilling than those who were planning or undecided about whether to get vaccinated. This indicates that lower-educated Evangelicals who have not been vaccinated may be another segment still open to a future decision to get a COVID-19 vaccine.

Findings offer insight into the health beliefs of Evangelical Christians. Consistent with the HBM, and when controlling for multiple variables, such as education, gender, age, race, health insurance, and rural status, those reporting a high level of benefits related to the COVID-19 vaccine, as well as those reporting a low level of barriers to getting the COVID-19 vaccine, were more likely to be vaccinated. Neither perceived severity of COVID-19 or perceived susceptibility to COVID-19 were significant predictors for COVID-19 vaccine uptake status. Therefore, communication strategies should focus on both increasing perceived benefits of the vaccine, and on adequately addressing perceived barriers to the vaccine.

The influence of Evangelical clergy on COVID-19 vaccine uptake presents a distinct opportunity for positive change [24]. The findings here showed that those who were receptive to faith-based vaccination promotion strategies were more likely to be vaccinated. This suggests that positive attitudes towards vaccination can be cued and reinforced by trusted religious leaders who themselves acknowledge getting the vaccine and encourage others to do the same or that clergy can be helpful in dealing with perceived barriers. Making churches vaccine distribution sites and helping parishioners get vaccine appointments could also be ways for evangelical communities to support higher uptake within their congregations. However, our findings also indicated that Evangelicals who reported seeking information from their religious leaders about getting the COVID-19 vaccine were significantly less likely to be vaccinated. Taken together, these findings indicate that faith-based strategies are helpful, but that seeking personal advice from Evangelical leaders may counteract these efforts. An open question for further research is the nature of the advice that Evangelical leaders provide when approached about COVID-19 vaccination. Working with Evangelical leaders so that they understand, endorse, and communicate the benefits of COVID-19 vaccination and its alignment with Christian values should continue to be a strategy for public health professionals and healthcare providers [24].

A hopeful result was that those who reported that their healthcare provider asked them about getting the COVID-19 vaccine were significantly more likely to be vaccinated; this could be an indication of these respondents trusting healthcare providers’ advice. This finding makes sense within the greater context of research demonstrating that trust in healthcare providers relates to improved health outcomes or behaviors [25]. This could also indicate a greater need for communication recommendations for providers to reach patients who rely heavily on religion for health decision making so that they can frame their recommendations in ways that are congruent with those value systems. However, among those who were not vaccinated, a healthcare provider talking to participants about vaccination was more associated with being unwilling to be vaccinated rather than planning to. This is likely because vaccination-unwilling participants had providers who tried to convince them unsuccessfully to change their mind.

An important contribution of these findings lies in the comparisons among the three unvaccinated groups, where consistent demographic results emerged, and findings indicated important health beliefs and faith-based considerations for communication with those who are undecided and planning to get the COVID-19 vaccine. Our analyses explored the differences between both those who were still undecided and those who were still planning to get the COVID-19 vaccine but had not yet received it, and those who did not plan to get the COVID-19 vaccine. These comparisons are important because a greater possibility remains for communication with Evangelicals who are planning or undecided to get vaccinated. When it comes to demographics, our results comparing unvaccinated individuals indicate that we must work diligently to reach those who have a higher level of formal education and live in suburban areas, as there may be a greater chance of successful communication with those who are less educated and in urban areas. Regarding health beliefs, our results indicate that health communication strategies related to COVID-19 vaccination for those who are either still undecided or planning to get the vaccine should focus on providing information both relating to perceived benefits as well as adequately addressing perceived barriers to the COVID-19 vaccines. Addressing these health beliefs amongst unvaccinated Evangelicals may help to prevent those who are still open to considering the vaccine from becoming entrenched in a position unwilling to get the vaccine, something that other studies caution can become the case [26]. Multinomial regression results also affirm the importance of healthcare providers addressing getting the COVID vaccine with their Evangelical patients. These conversations could be particularly helpful for those stating they are still planning to get the vaccine, especially for Evangelicals whose congregation is supportive of vaccination efforts.

This study has several strengths: it is one of the relatively few studies entirely focused on Evangelical Christians, a population with lower COVID-19 vaccine uptake levels. In addition, the results were analyzed through the lens of the HBM, a health behavior theory often used in studies of preventive behaviors such as vaccine uptake. In doing so, this study is at the intersection of religious studies and public health research, providing a unique lens to address a relevant public health and social phenomenon.

One of the main limitations of this study is potential selection bias. Individuals who are willing to complete an online survey (therefore, contribute to research) likely have a higher level of education and more openness to science [27]. As a result, some of the most vaccine-hesitant Evangelicals would likely not have participated in this study. Another limitation is that we treated Evangelicals as one relatively homogenous group, despite significant diversity within the movement. The identification of Evangelicalism has become more difficult since the 2016 presidential election, with some White voters increasing their identification with the label [15], while there has also been an increase in the number of “exvangelicals,” or former Evangelicals [28], many of whom might theologically resemble Evangelicals but not formally affiliate with the movement. There is also the question of race, where patterns of Evangelical affiliation are different among White, Black, Hispanic, and Asian Americans, who also have differing health outcomes related to their religious beliefs and practices [29]. Whereas our study was limited to examining Whites versus racial minorities because of sample size constraints, future work should investigate vaccination uptake differences in quota samples of Evangelicals from racial subgroups. Moreover, theological diversity abounds, such as differences between Pentecostals, who tend to look theologically similarly to Evangelicals (e.g., Assemblies of God, Church of God in Christ), and other Evangelical groups (e.g., Southern Baptist Convention, Presbyterian Church of America). Future studies should consider different types within Evangelicalism, such as classical Pentecostals (e.g., Assemblies of God), independent charismatics (e.g., Vineyard churches), Evangelicals located within mainline denominations (e.g., Evangelical Methodists), and more left-leaning Evangelicals (e.g., progressive Evangelicals). Furthermore, qualitative studies should be conducted amongst these groups to determine nuanced vaccine decision-making factors [30]. In addition, this study exclusively focused on self-identified Evangelicals, and did not include other religious groups, something that future studies should consider. Doing so would permit future studies to test to what extent Evangelicals are different regarding the effects of the tested independent variables and controls in comparison with other religious groups. Further, this exploratory study’s sample was not representative—a representative sample of Evangelicals is needed in order to validly transfer the results to the full population of all Evangelicals in the U.S. Additionally, the sample had a larger proportion of women than men, and no non-binary respondents were part of the sample. Although these unequal proportions are common in survey research [31], caution should be exerted when interpreting these findings as they are exploratory in nature, and claims about Evangelical men must be probed in future studies. Future studies should consider recruiting a more balanced sample by working with community partners. Finally, since studies have shown specific religious groups playing a similar role in vaccine acceptance and hesitancy [32], future studies should consider studying conservative religious groups outside of the U.S.

## 5. Conclusions

This study explored differences in demographics, health beliefs, and several faith-based variables among Evangelicals who had already received one of the COVID-19 vaccines, those who reported not being willing to get a COVID-19 vaccine, those who were still undecided, and those who were planning to get the vaccine. In addition to beliefs about the benefits and barriers to vaccination, the role of healthcare providers discussing vaccination with their Evangelical patients were important predictors of vaccination status. Information seeking from clergy as well as faith-based support are important factors that can influence vaccination status. Intervention efforts that account for demographics identified here, such as age, parental status, and location, that capitalize on partnerships between health providers and clergy in supportive congregations may be able to reach undecided Evangelicals. Overall, this study sheds light on the vaccine beliefs and barriers of self-identified Evangelicals and provides a foundation for future studies aimed at improving vaccine uptake within this population. To achieve optimal levels of vaccination in the United States, vaccination behaviors must be understood not only within the overall public health context but also within specific religious and faith-based contexts.

## Figures and Tables

**Table 1:** HBM and faith-based variables (N = 531).

Characteristic	Item	Questions	Likert Scale	Mean, SD	Cronbach’s Alpha
HBM	Perceived benefits	Getting a COVID-19 vaccination helped me feel less worried about getting COVID-19.	Strongly disagree, 1, to strongly agree, 7	4.2, 1.57	0.82
		Getting a COVID-19 vaccination has decreased my chance of getting COVID-19 or its complications.	Strongly disagree, 1, to strongly agree, 7		
		Now that I have gotten the COVID-19 vaccine, I will decrease the frequency of having to consult my doctor.	Strongly disagree, 1, to strongly agree, 7		
		Now that I have gotten the COVID-19 vaccine, I will not get sick from COVID-19.	Strongly disagree, 1, to strongly agree, 7		
		Now that I have gotten the COVID-19 vaccine, I help protect those around me from COVID-19.	Strongly disagree, 1, to strongly agree, 7		
HBM	Perceived barriers	I could not be bothered to get the COVID-19 vaccination.	Strongly disagree, 1, to strongly agree, 7	3.4, 1.16	0.81
		I am scared of needles.	Strongly disagree, 1, to strongly agree, 7		
		I am concerned about the side effects of the COVID-19 vaccination.	Strongly disagree, 1, to strongly agree, 7		
		The COVID-19 vaccine is expensive.	Strongly disagree, 1, to strongly agree, 7		
		There is a shortage of the COVID-19 vaccine.	Strongly disagree, 1, to strongly agree, 7		
		It is inconvenient to get the COVID-19 vaccination.	Strongly disagree, 1, to strongly agree, 7		
		Short-cuts have been taken to develop the COVID-19 vaccine quickly.	Strongly disagree, 1, to strongly agree, 7		
		Scientists did not have enough time to assess the possible risks of a COVID-19 vaccine.	Strongly disagree, 1, to strongly agree, 7		
		You might be required to get the COVID- 19 vaccine even if you don’t want to	Strongly disagree, 1, to strongly agree, 7		
		It will be difficult for you to travel to a vaccination site to get the COVID-19 vaccine	Strongly disagree, 1, to strongly agree, 7		
		You won’t be able to get the vaccine from a place you trust	Strongly disagree, 1, to strongly agree, 7		
		You might need to miss work if the side effects of the vaccine make you feel sick for a day or more	Strongly disagree, 1, to strongly agree, 7		
		You might need to take time off work to go and get the COVID vaccine	Strongly disagree, 1, to strongly agree, 7		
		The COVID-19 vaccine may negatively impact your fertility in the future	Strongly disagree, 1, to strongly agree, 7		
		The COVID-19 vaccines are not as safe as they are said to be	Strongly disagree, 1, to strongly agree, 7		
		The COVID-19 vaccines are not as effective as they are said to be	Strongly disagree, 1, to strongly agree, 7		
HBM	Perceived severity	Complications from COVID-19 are serious.	Strongly disagree, 1, to strongly agree, 7	4.8, 1.51	0.76
		I will be very sick if I get COVID-19.	Strongly disagree, 1, to strongly agree, 7		
		I am afraid of getting COVID-19.	Strongly disagree, 1, to strongly agree, 7		
HBM	Perceived susceptibility	My chance of getting COVID-19 in the next few months is high.	Strongly disagree, 1, to strongly agree, 7	3.8, 1.46	0.81
		I am worried about the likelihood of getting COVID-19 in the near future.	Strongly disagree, 1, to strongly agree, 7		
		Getting COVID-19 is currently a possibility for me.	Strongly disagree, 1, to strongly agree, 7		
HBM	Self-efficacy	If I wanted to, I am confident that I could get the COVID-19 vaccination.	Strongly disagree, 1, to strongly agree, 7	5.7, 1.26	0.77
		How certain are you that you could get the COVID-19 vaccination?	Very uncertain, 1, to very certain, 7		
		For me, getting a COVID-19 vaccination would be…	Very difficult, 1, to very easy, 7		
HBM	Cues to action	Has a healthcare provider spoken to you about getting the COVID-19 vaccine?	Yes, 1, or No, 0	N/A	N/A
Faith-based	Advice from religious leader	When deciding whether to get a COVID-19 vaccine, how much did you turn to a religious leader such as minister or pastor for information?	Not at all, 1, to a lot, 4	2.3, 1.07	N/A
Faith-based	Faith-based support	A religious leader you trust encouraged you to get the vaccine	Much less likely, 1, to much more likely, 5	3.4, 0.99	0.84
		You could get the vaccine at a nearby religious congregation	Much less likely, 1, to much more likely, 5		
		A religious leader you trust got the vaccine	Much less likely, 1, to much more likely, 5		
		Your religious community provided assistance in getting an appointment to get the vaccine	Much less likely, 1, to much more likely, 5		

**Table 2:** Descriptives of the overall sample (N = 531).

Characteristics	% (*n*)
Education	
Bachelor’s degree or higher	35.6% (*n* = 189)
Some college	39.2% (*n* = 208)
High school diploma/some high school but no diploma	25.2% (*n* = 134)
Gender	
Man	35.2% (*n* = 187)
Woman	64.8% (*n* = 344)
Non-binary	0% (*n* = 0)
Age, years	
Mean, SD	51.2, 16.5
Race/ethnicity	
White	82.1% (*n* = 436)
Non-White	17.9% (*n* = 95)
Rural/Urban/Suburban	
Rural	32.4% (*n* = 172)
Suburban	36.5% (*n* = 194)
Urban	31.1% (*n* = 165)
Parent of child <18, living at home	
Yes	36.7% (*n* = 195)
No	63.3% (*n* = 336)
COVID-19 vaccine status	
One or more doses	40.5% (*n* = 215)
Planning to get vaccine	19.6% (*n* = 104)
Undecided	20.3% (*n* = 108)
Unwilling to get vaccine	19.6% (*n* = 104)
Health insurance	
Yes	90.4% (*n* = 480)
No	9.6% (*n* = 51)
Health Belief Model	
Cues to action (provider contact = yes)	64.0% (*n* = 340)
Health Belief Model	*M, SD*
Perceived benefits of the COVID-19 vaccine	4.16, 1.57
Perceived barriers to the COVID-19 vaccine	3.43, 1.16
Perceived severity of COVID-19	4.85, 1.51
Perceived susceptibility of COVID-19	3.80, 1.46
Self-efficacy	5.66, 1.26
Faith-based variables	
Faith-based support	3.41, 0.99
Advice from clergy	2.29, 1.07

**Table 3:** Logistic regression predicting COVID-19 vaccine uptake (N = 531).

Variable	*p*-Value	OR (95% CI)
Age *	0.001	1.04 (1.02, 1.06)
Gender: woman (Ref.: man)	0.714	0.90 (0.49, 1.62)
Education: some HS/HS diploma (Ref.: Bachelor’s)	0.219	0.63 (0.30, 1.32)
Education: some college (Ref.: Bachelor’s)	0.346	1.44 (0.67, 3.08)
Rurality: suburban (Ref.: rural) *	0.019	2.26 (1.14, 4.48)
Rurality: urban (Ref.: rural)	0.978	0.99 (0.42, 2.30)
Race: White (Ref.: non-White)	0.657	1.18 (0.57, 2.44)
Health insurance (Ref.: no health insurance)	0.792	0.87 (0.32, 2.38)
Parent (Ref.: No) *	0.009	0.37 (0.17, 0.78)
HBM: Severity	0.642	0.95 (0.78, 1.17)
HBM: Susceptibility	0.907	1.01 (0.82, 1.26)
HBM: Benefits *	<0.001	1.80 (1.38, 2.37)
HBM: Barriers *	<0.001	0.36 (0.25, 0.51)
HBM: Self-efficacy	0.143	0.83 (0.64, 1.07)
HBM: Cues to action/health provider contact (Ref.: no provider contact) *	0.027	2.01 (1.08, 3.75)
Religious leader information seeking *	<0.001	0.42 (0.31, 0.57)
Faith-based support for vaccination *	0.007	1.61 (1.14, 2.27)

* *p* < 0.05.

**Table 4:** Binary logistic regression: predictors of non-vaccination status (n = 316).

	*p*-Value Undecided vs. Will Not Get Vaccinated	Undecided vs. Unwilling to Get Vaccinated, OR (95% CI)	*p*-Value Planning vs. Will Not Get Vaccinated	Planning vs. Unwilling to Get Vaccinated OR (95% CI)
Age	0.423	0.99 (0.96, 1.02)	0.173	0.97 (0.93, 1.01)
Gender: women (Ref.: man)	0.644	1.29 (0.58, 2.89)	0.673	0.84 (0.28, 2.56)
Health insurance (Ref.: no)	0.453	0.67(0.22, 2.09)	0.243	2.75 (0.52, 14.48)
Parent (Ref.: no)	0.622	1.27 (0.50, 3.25)	0.688	0.76 (0.20, 2.86)
Education: some HS/HS diploma (Ref.: Bachelor’s)	0.046	0.35 (0.12, 0.98)	0.002	0.09 (0.02, 0.44)
Education: some college (Ref.: Bachelor’s)	0.807	0.91 (0.35, 2.37)	0.473	0.64 (0.19, 2.17)
Race/Ethnicity: White (Ref.: minority)	0.261	1.82 (0.58, 5.66)	0.169	2.68 (0.65, 11.03)
Rurality: rural (Ref.: urban)	0.619	0.81 (0.27, 2.42)	0.581	0.71 (0.16, 3.06)
Rurality: suburban (Ref.: urban)	0.055	0.34 (0.11, 1.02)	0.030	0.21 (0.05, 0.85)
HBM: Severity	0.995	0.99 (0.69, 1.43)	0.136	0.68 (0.41, 1.13)
HBM: Susceptibility	0.056	1.39 (0.98, 1.96)	0.002	2.15 (1.33, 3.47)
HBM: Benefits	<0.001	2.11 (1.45, 3.06)	<0.001	4.54 (2.64, 7.84)
HBM: Barriers	0.013	0.39 (0.19, 0.83)	0.001	0.22 (0.087, 0.53)
HBM: Self-efficacy	0.334	1.29 (0.58, 2.89)	0.756	1.08 (0.66, 1.75)
HBM: Cues to action (health provider contact) (Ref.: no provider contact)	0.565	0.78 (0.37, 1.67)	0.024	0.26 (0.08, 0.84)
Faith-based support for vaccination	0.155	1.71 (0.83, 3.53)	0.036	2.58 (1.07, 6.22)
Religious leader information seeking	0.490	1.23 (0.82, 1.85)	0.819	1.09 (0.61, 1.96)

## Data Availability

The data presented in this study are available on request from the corresponding author.
